# Durable Response to Cemiplimab in Advanced Cutaneous Squamous Cell Carcinoma With Extensive Gluteal and Sacral Bone Infiltration: A 12-Month Case

**DOI:** 10.7759/cureus.86507

**Published:** 2025-06-21

**Authors:** Ivan Bivolarski

**Affiliations:** 1 Medical Oncology, Integrated Oncology Centre, Burgas, BGR

**Keywords:** bone metastases, cemiplimab, gluteal tumor, immunotherapy, sacral infiltration, squamous cell carcinoma of the skin

## Abstract

Cutaneous squamous cell carcinoma (cSCC) is a common skin malignancy, which, in its advanced stages, may involve regional lymph nodes and osseous structures, resulting in poor prognosis. Massive tumor infiltration of the sacrum and pelvic bones is rare and typically associated with limited treatment options and unfavorable outcomes. We present the case of a 59-year-old male with advanced cSCC involving the gluteal region, sacrum, pelvic lymph nodes, and bones. As part of a personalized treatment approach, the patient received a combination of cemiplimab, a PD-1 checkpoint inhibitor, and denosumab for bone protection. Despite the aggressive nature and anatomical complexity of the disease, the patient demonstrated a marked and durable clinical response, with radiographic evidence of partial metabolic regression on PET/CT (SUVmax reduction from 9.87 to 6.37), resolution of sacral pain, and full recovery of ambulatory function. No serious immune-related toxicities were observed. This case illustrates the potential of integrating immunotherapy with supportive bone-targeted therapy to achieve meaningful disease control in rare presentations of bone-invasive cSCC.

## Introduction

Cutaneous squamous cell carcinoma (cSCC) is one of the most common non-melanoma skin cancers, accounting for approximately 20% of all cutaneous malignancies [1]. Although most cSCCs are curable when treated early, advanced or metastatic forms -occurring in fewer than 5% of cases - are associated with poor prognosis and limited treatment options [2].

Clinically, cSCC often presents as an erythematous, keratotic, or ulcerated lesion in sun-exposed areas, such as the face, scalp, ears, and extremities. Key risk factors include cumulative ultraviolet radiation exposure, immunosuppression, older age, and prior skin injury or malignancy [3].

Extensive disease involving the gluteal region, pelvic lymph nodes, and sacral or pelvic bones is exceptionally rare and presents unique diagnostic and therapeutic challenges. Osseous invasion is a negative prognostic factor associated with reduced survival and poor response to conventional therapies.

Recent clinical evidence has emphasized the need for novel therapeutic strategies in advanced cSCC. The pivotal EMPOWER-CSCC-1 trial demonstrated that cemiplimab - a high-affinity, fully human monoclonal antibody targeting the programmed death-1 (PD-1) receptor - can improve overall survival and induce durable responses by restoring antitumor T-cell activity [3].

In cases with bone involvement, combining cemiplimab with denosumab, a RANKL inhibitor used to prevent skeletal-related events, may offer additional benefit by stabilizing the affected bone structure and reducing tumor-driven osteolysis.

We present a rare case of advanced cSCC with sacral and pelvic bone infiltration, successfully managed through this combined immuno-skeletal approach. Sustained clinical and metabolic response was achieved and confirmed by serial PET/CT imaging. This case illustrates the importance of personalized, interdisciplinary strategies in addressing complex oncologic presentations.

## Case presentation

A 59-year-old male was diagnosed with advanced cSCC of the gluteal region, with infiltration of the sacrum, pelvic lymph nodes, and pelvic bones. The lesion had initially been misdiagnosed as a dermoid cyst and had been under observation for several years without definitive treatment. In 2023, the patient reported worsening pain in the sacral and pelvic area, prompting a biopsy. Histopathological analysis confirmed the diagnosis of cSCC.

On November 9, 2023, the patient underwent sequestrectomy, curettage, and surgical revision of the sacral bone. The tumor involved skin, soft tissue, and bone. Clinical staging was rT4 N1 M1 (oss, ski, oth), Eastern Cooperative Oncology Group (ECOG) performance status 1, stage IV. PD-L1 expression was not assessed.

**Figure 1:**
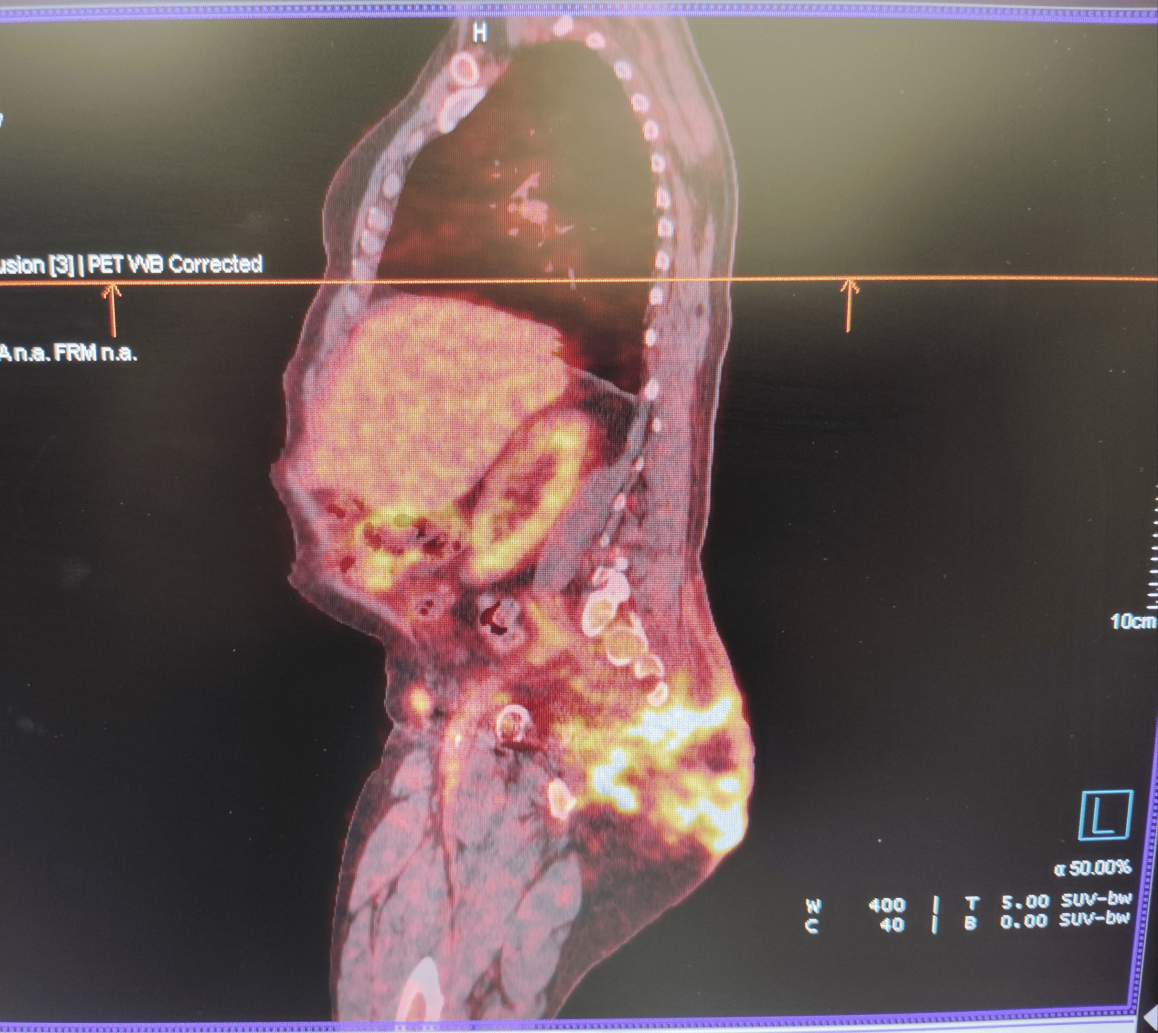
Baseline PET/CT scan performed in December 2023, prior to the initiation of cemiplimab and denosumab, showing extensive hypermetabolic tumor infiltration in the gluteal region and sacrum.

A PET/CT scan performed in December 2023 revealed a bulky hypermetabolic lesion infiltrating the right sacral wing and pararectal tissues (SUVmax: 9.87), as well as metabolically active pelvic and bilateral inguinal lymph nodes (SUVmax: up to 3.74) (Figure 1). No distant organ metastases were identified.

**Figure 2:**
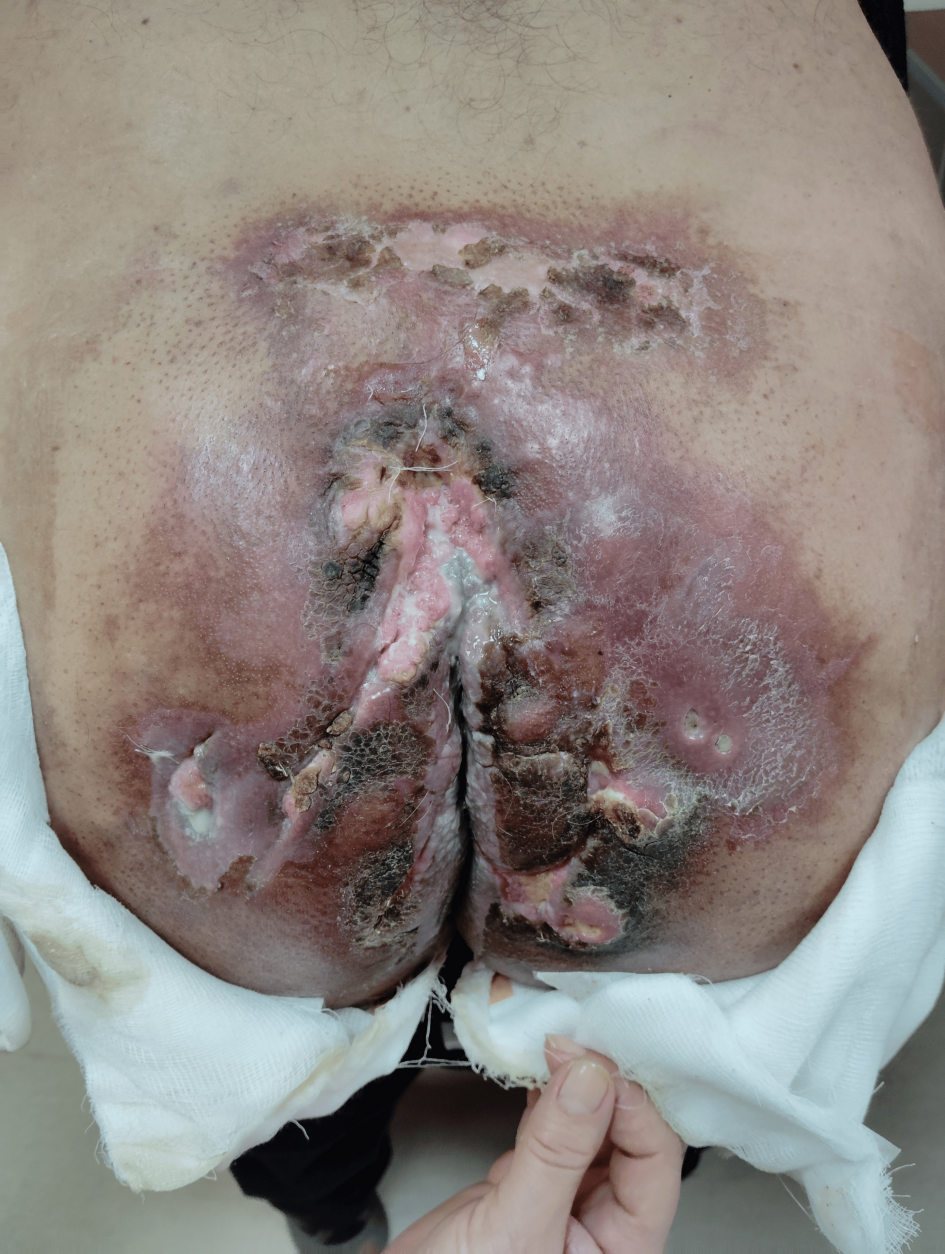
Clinical image of the gluteal lesion taken in February 2024, shortly before treatment initiation, demonstrating extensive ulceration, necrosis, and inflammation affecting the sacral and perianal region.

Between January 4 and February 7, 2024, the patient received palliative radiotherapy to the sacral region for pain control. Subsequently, a combined treatment with cemiplimab (administered every 21 days) and denosumab (every 28 days) was initiated on February 27, 2024 (Figure 2). Cemiplimab was consistently administered in the late morning, between 10:00 AM and 12:00 PM. Denosumab was scheduled exactly one week after each cemiplimab cycle and administered during the same time window.

This temporal coordination was maintained throughout the course of therapy as part of a structured, personalized treatment strategy, aiming to optimize physiological synchrony and tolerability.

On March 22, 2024, due to progressive anemia (hemoglobin: 97 g/L), epoetin alfa (10,000 IU subcutaneously) was initiated. The patient responded well, and denosumab continued to support local bone control. Hemoglobin levels remained stable in the range of 84-101 g/L despite ongoing bone infiltration.

Leukocyte counts ranged from 22 to 30 × 10⁹/L throughout treatment, yet no immune-related adverse events (irAEs) were observed.

**Figure 3:**
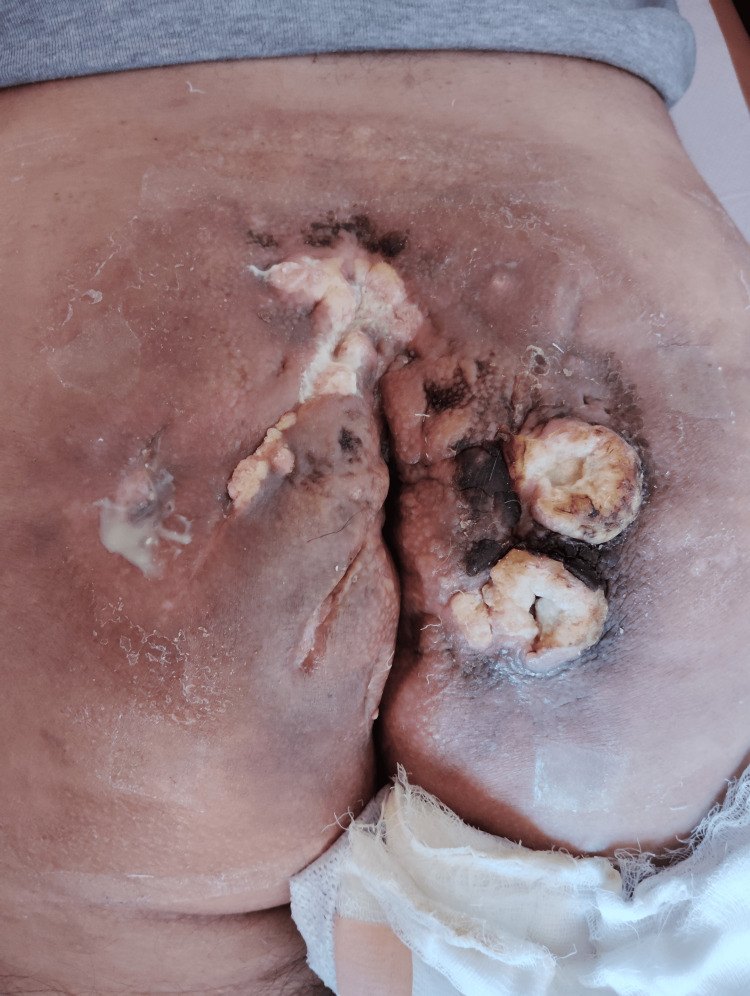
Clinical image taken in May 2024 after three cycles of cemiplimab, demonstrating partial epithelialization and reduced peripheral inflammation compared to baseline. Necrotic tissue remains present, but local control has improved.

**Figure 4:**
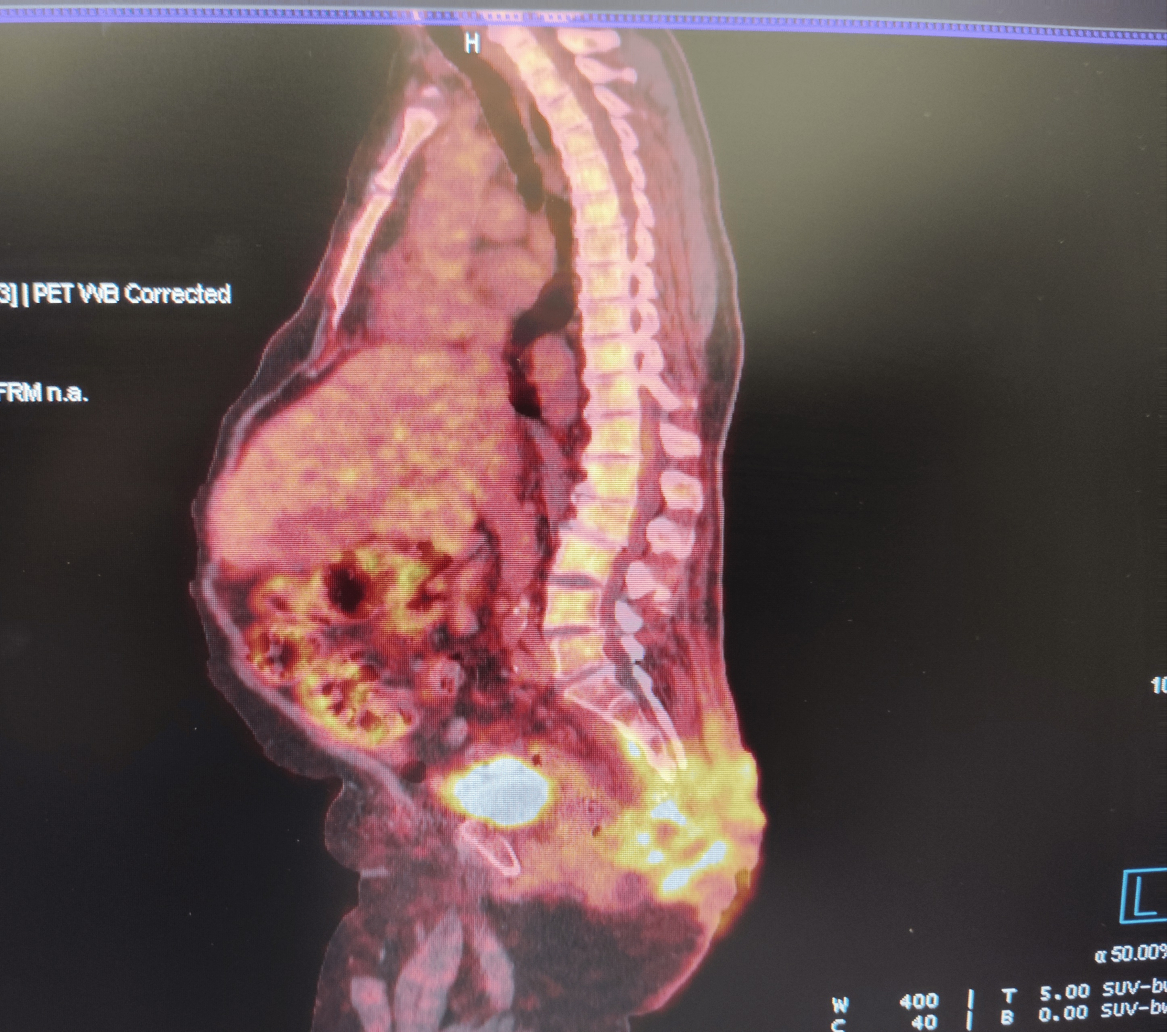
PET/CT performed in May 2024, three months after treatment initiation, showing significant reduction in metabolic activity and partial tumor regression in the sacral and gluteal areas.

In May 2024, a follow-up PET/CT confirmed sustained partial metabolic response. The SUVmax of the primary sacral lesion had decreased to 6.37 (previously 9.87), while bilateral inguinal lymph nodes remained metabolically stable with SUVmax up to 4.11. No new hypermetabolic lesions were observed (Figure 3, Figure 4).

Imaging on July 12, 2024, identified an infiltrative gluteal lesion and a secondary hypermetabolic focus at the base of the right pelvic wing. A follow-up MRI on August 14, 2024, confirmed these findings.

Due to persistent sacral and pelvic pain, an initial analgesic regimen with Viktanil 100 mg was prescribed. This was subsequently revised to include Oxilan 40 mg and Sublifen, providing better pain control. Topical and systemic metronidazole was also introduced to manage localized inflammation and reduce bacterial load.

**Figure 5:**
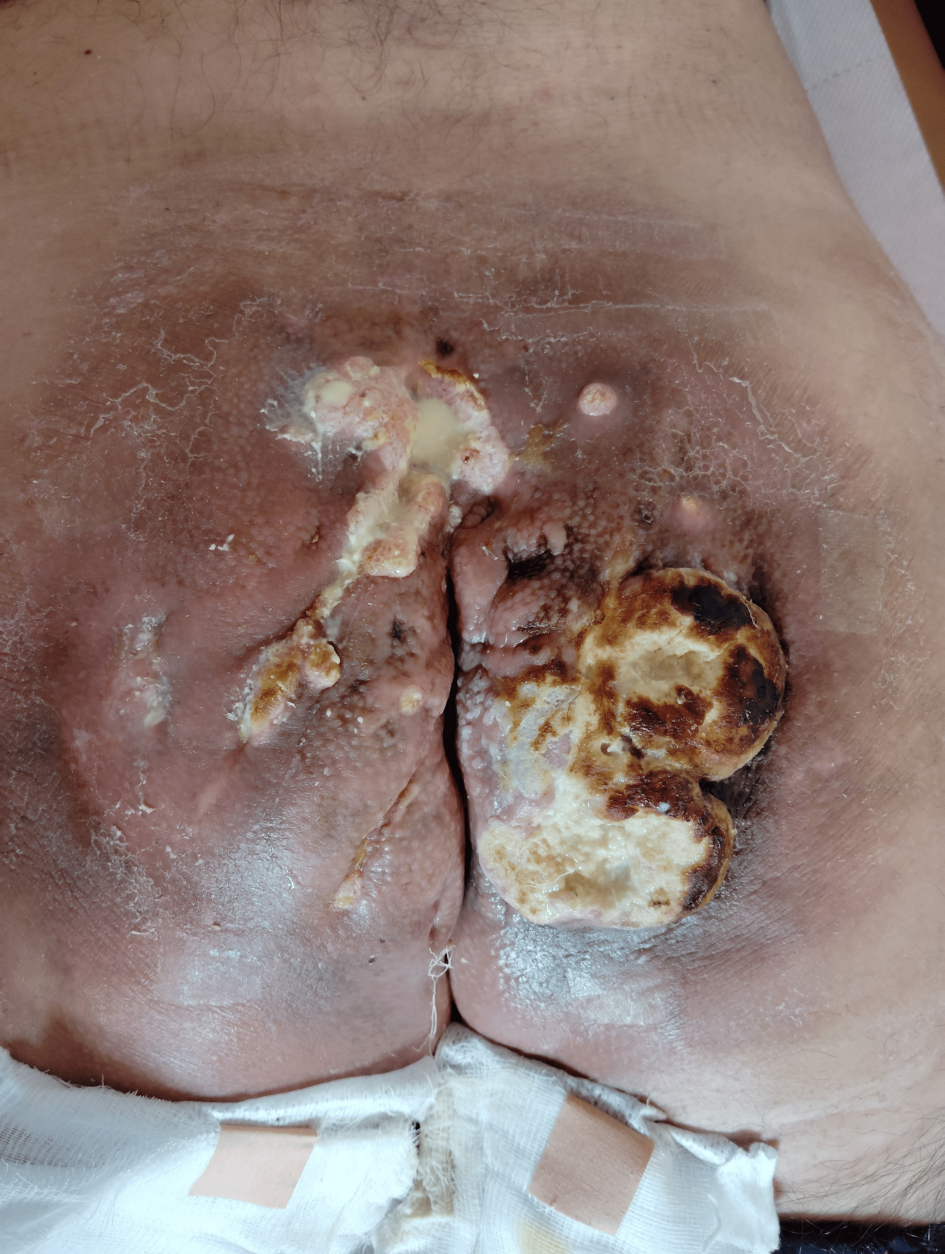
Gluteal region image (August 2024) showing residual soft tissue inflammation and subcutaneous irregularity near the sacral area, with improved surface integrity following combined systemic therapy and local supportive treatment.

The patient continued cemiplimab immunotherapy and denosumab therapy throughout August and September 2024, with good tolerability. Metronidazole was maintained as an adjunctive measure to address local tissue irritation and minimize infection risk (Figure 5).

Despite the advanced stage and anatomical complexity of the disease, the patient demonstrated a durable clinical response, with tumor regression and improved symptom control. He remains under active surveillance and continues to receive cemiplimab and denosumab according to the established schedule. As of the current report, he has completed nine cycles of cemiplimab and eight doses of denosumab.

**Figure 6:**
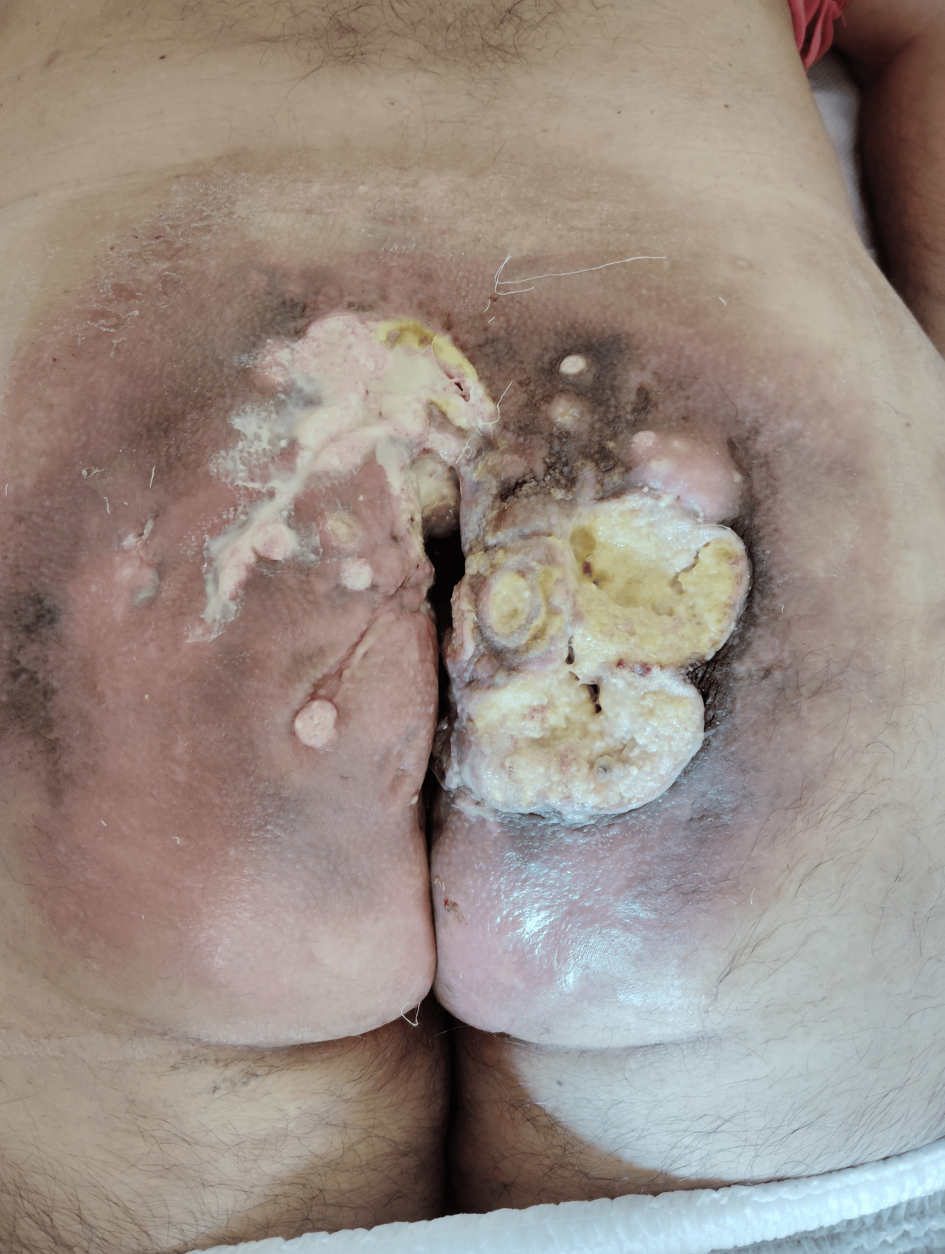
Clinical image from late October 2024, showing further ulcer progression, necrosis, and exudate accumulation. These findings prompted intensified local management and continued use of metronidazole.

The patient currently reports improved functional capacity and daily comfort compared to baseline, with stable mobility and reduced pain levels (Figure 6). This case illustrates the feasibility of combining checkpoint inhibition and bone-targeted therapy in anatomically complex, late-presenting cSCC, while highlighting the potential benefit of maintaining temporal consistency in treatment administration. Following the most recent clinical and imaging evaluations, a multidisciplinary tumor board recommended continuation of the current regimen - cemiplimab in combination with denosumab - until evidence of clinical progression or unacceptable toxicity emerges.

## Discussion

Metastatic cSCC is associated with a poor prognosis, particularly in cases involving extensive skin lesions and bone infiltration. Such presentations are not only rare but also represent significant therapeutic challenges due to limited data, rapid disease progression, and poor response to conventional treatment [1-3].

Historically, systemic therapy for advanced cSCC was based primarily on platinum-based chemotherapy regimens, such as carboplatin and paclitaxel, which showed limited efficacy and considerable toxicity [2,3]. While these agents provided transient responses, they failed to significantly improve long-term survival outcomes, particularly in frail or elderly patients [4,5]. This treatment gap underscores the need for more effective and better-tolerated therapeutic strategies.

The advent of immunotherapy, particularly immune checkpoint inhibitors (ICIs), has revolutionized the management of advanced cSCC. Cemiplimab, a fully human monoclonal antibody targeting PD-1, was the first ICI approved specifically for advanced cSCC. The pivotal EMPOWER-CSCC-1 trial demonstrated significant and durable response rates, along with improved survival and quality of life [6,7]. Similar results have been observed with other ICIs, including pembrolizumab, in both squamous and non-squamous skin cancers [8-10].

Nonetheless, achieving effective disease control remains particularly challenging in cases with osseous involvement. Bone metastases can increase the risk of pathological fractures, spinal cord compression, and hypercalcemia, which collectively reduce functional independence and quality of life. In such cases, bone-targeted therapies such as denosumab, a RANKL inhibitor, have a well-established role in reducing skeletal-related events [5,11]. Moreover, emerging evidence suggests that RANKL inhibition may modulate the tumor microenvironment and enhance the efficacy of PD-1 blockade [12,13].

In the present case, the integration of cemiplimab and denosumab into a personalized treatment regimen resulted in a significant clinical response, including reduction of tumor burden and improved symptom control. The uniqueness of the case - marked by extensive gluteal, sacral, and pelvic bone involvement - emphasizes the need for individualized, interdisciplinary management strategies. Clinical staging and radiologic assessment played an essential role in guiding the therapeutic course [7,8,14].

Effective pain management also emerged as a critical element of care. Analgesic strategies were tailored to evolving symptoms and complemented the oncologic treatment. This reinforces the importance of integrating supportive and palliative care measures early in the treatment plan [8,9,15].

Future studies should investigate optimal combinations of immunotherapy, radiotherapy, and bone-modifying agents in advanced cSCC. Registry-based analyses and real-world data will be invaluable for validating current approaches and identifying predictors of long-term benefit [13-16].

In this context, consistent timing of immunotherapy and supportive treatments, as applied in our case, may also warrant further exploration as a potential modifier of treatment tolerance and efficacy. Although evidence remains preliminary, emerging chronotherapeutic models suggest that synchronizing systemic therapy with circadian immune dynamics may improve outcomes in select cancer types [17-19].

## Conclusions

This clinical case illustrates that immunotherapy with cemiplimab, combined with denosumab for bone protection, can serve as an effective and well-tolerated treatment strategy for advanced cSCC with extensive sacral and pelvic bone involvement. Despite diagnostic delay and anatomically complex disease progression, the patient achieved a sustained clinical response, with tumor regression, pain control, and functional improvement.

Importantly, this case highlights the value of personalized and multidisciplinary care, integrating oncologic, orthopedic, radiologic, and supportive strategies. The structured temporal coordination of systemic therapy, maintained consistently throughout treatment, may have contributed to favorable tolerability and therapeutic synchrony. Future clinical investigations should explore optimized immunotherapy-based regimens, including the role of bone-targeted agents and chronotherapeutic timing, in the management of high-risk, bone-invasive cSCC.
